# The nature of science in Chinese senior secondary geography textbooks under the context of sustainable development: Identifying the gaps and seeking reform

**DOI:** 10.1371/journal.pone.0357310

**Published:** 2026-09-17

**Authors:** Weirui Zhang, Xiangdong Wang, Changhong Wu, Chenlong Dong

**Affiliations:** 1 Faculty of Education, Northeast Normal University, Changchun, China; 2 College of Geographical Sciences, Northeast Normal University, Changchun, China; 3 National Key Research Base for Textbook Research in Primary and Secondary School Science (Integrated), Ministry of Education, Northeast Normal University, Changchun, China; 4 Library, Northeast Normal University, Changchun, China; Nanjing University, CHINA

## Abstract

Within the context of sustainable development, UNESCO has recognized geography as a core discipline for advancing education for sustainable development. Addressing complex sustainability issues calls for geography education to make visible how geographical knowledge is produced, justified, communicated, and situated in social contexts, alongside the presentation of established scientific conclusions. Guided by the Family Resemblance Approach (FRA), this study uses Epistemic Network Analysis (ENA) to examine and visualize representations of the Nature of Science (NOS) in selected Chinese senior secondary geography textbooks. Across the corpus, Scientific Knowledge and Scientific Practice account for much of the frequency profile, and ENA identifies a recurrent C.A.V-C.SP-C.SK core structure. Social values are most evident where geographical knowledge is linked to practical applications, whereas professional activities, scientific norms, organizational interaction, and power-related dimensions are less consistently connected with these knowledge-practice relations. Viewed in relation to the disciplinary character of geography, the findings inform textbook development and curriculum reform by drawing attention to the representation of relationships among geographical knowledge, evidence, methods, values, institutions, and sustainability-oriented decision-making.

## 1. Introduction

In 2015, the United Nations adopted the 2030 Agenda for Sustainable Development and established 17 Sustainable Development Goals (SDGs) [1]. The targets have also been examined from a scientific perspective [2], while textbooks have been promoted as a means of embedding sustainability-related knowledge, values, and competencies in school curricula [3]. Geography has a close connection with Education for Sustainable Development (ESD) because it addresses human-environment relations and brings together the natural and social dimensions of sustainability [4]. Puttick and Cullinane describe the discipline through interacting dimensions of aims and values, knowledge, methods, practices, and social-institutional organization [5]. Geography textbooks thus provide a curricular setting in which sustainability-related knowledge, practices, values, and forms of reasoning can be represented.

Sustainability education also depends on how scientific knowledge is understood as a product of inquiry. Questions about how knowledge is produced, justified, communicated, constrained, and applied fall within the Nature of Science (NOS). NOS is widely regarded as an important component of scientific literacy [6], and scientific literacy includes forms of reasoning needed for meaningful engagement with science-related questions and decisions [7]. Sustainability problems in geography often have features of “wicked problems,” including uncertainty and contested definitions of the problem itself [8], while also involving ethical and political challenges [9]. The connection between NOS and geography is therefore rooted in the discipline’s own practices of knowledge production: geographical claims are developed through evidence, methodological choices, representations, explanations, and socially situated applications. Geography textbooks can be examined from this perspective because they give curricular form to these processes and to the epistemic and social conditions under which geographical knowledge is produced and used.

Research on NOS in textbooks has expanded considerably, although most studies still concern physics, chemistry, and biology, with geography receiving much less attention. One unresolved question is how geography textbooks represent the relationships among geographical knowledge production, methodological choices, evidence, social values, and institutional contexts, and whether these elements appear as connected parts of knowledge-making. The Family Resemblance Approach (FRA) conceptualizes science as an integrated cognitive-epistemic and social-institutional system, which makes it well suited to this relational question [10]. A systematic review documents the increasing use of FRA in science-education research, including curriculum and textbook studies [11]. On this basis, the present study combines FRA with Epistemic Network Analysis (ENA) to examine the distribution of NOS representations and the structure of their connections in selected Chinese senior secondary geography textbooks.

Research question 1: How are FRA-based NOS dimensions distributed across the selected Chinese senior secondary geography textbooks?

Research question 2: What structural patterns characterize relationships among NOS dimensions within and between the cognitive-epistemic and social-institutional systems?

Research question 3: What educational implications can be inferred from these distributional and structural patterns for scientific literacy and ESD-oriented geography textbook design?

The three questions move from description to relational analysis and then to interpretation. RQ1 examines the distribution of NOS dimensions, RQ2 addresses the structure of their relationships, and RQ3 considers the educational implications that can be inferred from these textual patterns for scientific literacy and ESD-oriented geography textbook design.

## 2. Literature review

### 2.1. Conceptual development of the nature of science

The Nature of Science (NOS) is included in major science education standards [12] and has remained a longstanding goal of science curricula [13]. Lederman [14] described NOS in terms of the values and assumptions underlying science and scientific knowledge, including creativity, provisionality, empirical evidence, subjectivity, testability, and the cultural and social embeddedness of science. The later “consensus view” was criticized for paying insufficient attention to differences among disciplines and for representing science in ways that could understate its changing character. Hodson and Wong [15], for example, called for a broader and richer treatment of NOS in curricula.

Irzik and Nola proposed a family resemblance account of NOS [16], later developed by Erduran and Dagher into the Family Resemblance Approach (FRA) [10]. FRA recognizes both shared features and disciplinary variation. In Erduran and Dagher’s extended formulation, NOS is conceptualized as an interconnected cognitive-epistemic and social-institutional system [10]. The FRA approach has been increasingly adopted in NOS teaching interventions, curriculum and textbook analysis, and studies of students’, teachers’, and experts’ understanding of NOS [11,17,18]. For curriculum and textbook research, FRA is useful because it treats NOS as a set of related dimensions within a broader system of scientific activity [10].

### 2.2. Research on NOS in science textbooks

Textbooks can shape how educational priorities are communicated to students and translated into classroom learning materials [19]. The representation of NOS in educational materials, particularly science textbooks, has garnered widespread academic attention. Abd-El-Khalick, Waters, and Le [20] found limited and often implicit NOS representations in 14 chemistry textbooks. Reinisch and Fricke [21] found that German biology textbooks emphasized cognitive-epistemic aspects over social-institutional dimensions. Abd-El-Khalick et al. [22] concluded that high school biology and physics textbooks lacked depth in NOS representation. Chiappetta and Fillman [23] found that the five U.S. high school biology textbooks they examined showed a more balanced representation of four science-literacy themes than comparable textbooks analyzed 15 years earlier, particularly through greater attention to investigation and how scientists work. Caramaschi et al. [24] found that Italian physics curriculum documents predominantly focused on the cognitive dimension with internal inconsistencies. Yeh et al. [25] found that Scientific Practices, Scientific Knowledge, and Social Values were the three most frequently represented RFN categories in the analyzed South African physical sciences textbooks. Cheung [26] analyzed Hong Kong biology curriculum and examination papers using the FRA framework. Li et al. [27] reported improvements in Chinese high school biology textbooks from 2004 to 2019, particularly in the frequency and quality of NOS representations, while also noting an imbalanced distribution with greater emphasis on empirical and tentative aspects. Upahi et al. [28] found that Nigerian chemistry textbooks typically presented NOS implicitly. Chua et al. [29] found imbalanced NOS representation in Singaporean biology textbooks. Bancong et al. [30] found that South Korean physics textbooks contained more NOS elements than Indonesian physics textbooks, especially in the cognitive–epistemic dimension, although both sets of textbooks gave limited attention to some important NOS aspects.

Across these studies, NOS representation is often uneven. Scientific knowledge, inquiry processes, and scientific practices generally receive more explicit treatment than the social and institutional conditions through which science is organized and situated in society. The presence of individual NOS elements also says little about whether a textbook presents them as parts of a connected epistemic and social enterprise. Scientific observations, experiments, or explanations may be included while the evaluation of evidence within scientific communities, the consequences of methodological choices, or the interaction between scientific knowledge, institutions, and social values receives much less attention. This pattern makes the organization and interconnection of NOS dimensions an important object of textbook analysis in addition to their presence and frequency.

Most NOS textbook research has concentrated on physics, chemistry, biology, and general science, leaving geography comparatively underexamined. The gap matters for theoretical as well as empirical reasons. Geography is an integrative discipline that studies natural and human environments, draws on perspectives from the natural and social sciences, and addresses spatial variability and human-environment relationships from local to global scales [31]. Geographical knowledge is produced through a wide range of evidence and inquiry practices, including observation, fieldwork, mapping, remote sensing, geographic information systems (GIS), statistical analysis, modelling, and regional investigation. These epistemic and methodological features need to be considered before findings from conventional science textbooks can be applied to geography.

Geography textbooks enter NOS research through the ways they represent geographical knowledge-making. Alongside established concepts, spatial patterns, and explanations, textbooks may show how geographical questions are formulated, how evidence is generated and interpreted, how methods and representations are chosen, how explanations are constructed, and how knowledge is applied to human-environment problems. They therefore provide a curricular setting for examining the epistemic, methodological, practical, and social dimensions of geographical knowledge. Puttick and Cullinane [5] offer a theoretical basis for this work by extending ideas from NOS and the Family Resemblance Approach to the Nature of Geography. Systematic evidence remains limited, however, on how these dimensions are represented in geography textbooks and, especially, how they are connected.

### 2.3. The Nature of Science in geography education

In geography education, broader NOS dimensions can be interpreted in relation to the disciplinary characteristics of geography. Drawing on the Family Resemblance Approach, Puttick and Cullinane [5] proposed the Nature of Geography framework, describing geography through its aims and values, knowledge, methods and methodological rules, practices, and social-institutional system. The development of geographical knowledge has also involved changing traditions of observation, representation, explanation, and social interpretation [32]. In this study, these ideas provide a basis for examining NOS in geography in terms of geographical knowledge claims and the processes through which they are produced, represented, justified, communicated, and used within particular spatial, methodological, and social contexts.

A central epistemic feature of geography is the spatial, regional, and scalar conditionality of knowledge. Geographical explanations ask where phenomena occur, why they occur in particular locations, how places and regions are connected, and whether relationships change across scales. Because the patterns identified and the explanations considered adequate may depend on the scale of analysis [33], geographical knowledge is often context-sensitive. Its evidential basis is similarly diverse, drawing on field observations, measurements, maps, statistical records, remote-sensing imagery, GIS data, and historical materials. These sources often need to be compared and integrated across locations, periods, and levels of resolution.

Geographical evidence is also mediated by representation and technology. Maps necessarily involve selection, simplification, projection, scale, classification, and symbolization, all of which affect what spatial patterns become visible and how they are interpreted [34]. GIS and related geospatial technologies further extend this epistemic role: geographical information is generated and analyzed through particular data models, resolutions, classifications, and procedures [35]. Contemporary data infrastructures likewise shape how data are organized, accessed, and interpreted [36]. From an NOS perspective, maps and geospatial technologies are therefore not neutral presentation tools but part of the process through which geographical evidence and explanations are constructed.

Geographical inquiry is characterized by methodological plurality and problem-dependent methodological choice. Fieldwork, measurement, experimentation, statistical and spatial analysis, modelling, comparison, interviews, and documentary analysis may all contribute to geographical inquiry. Enquiry-oriented geography education similarly emphasizes the formulation of questions and the active investigation of evidence [37]. No single procedure can therefore represent “the geographical method”; methodological choices depend on the research question, available evidence, spatial and temporal scale, and explanatory purpose. This plurality is closely related to geographical explanation, in which concepts, hypotheses, theories, laws, and models are used to construct and evaluate accounts of spatial phenomena [38]. Because models depend on assumptions, data quality, parameters, boundaries, and scale, model outputs should be interpreted as mediated representations rather than direct reproductions of reality.

Geographical explanation is also integrative. Environmental change, natural hazards, resource use, population change, and urban or regional development usually involve interactions among physical processes, human activities, and spatial conditions. Human-environment relationships therefore provide an important epistemic context in which multiple forms of evidence, different scales, and natural and social explanations must be synthesized. This integrative orientation also connects geographical knowledge to environmental management, land-use planning, resource allocation, disaster-risk reduction, and other forms of public decision-making. In such contexts, scientific evidence may inform several possible actions rather than determine a single solution, because institutional responsibilities, social priorities, economic interests, ethical considerations, and competing values may also shape decisions.

These features make the social-institutional dimensions of NOS especially relevant to geography. The Nature of Geography framework treats geography as a cognitive-epistemic practice embedded in a social-institutional system [5], and geographical education research likewise attends to the relationships among disciplinary knowledge, educational practice, and wider forms of social interaction [39]. General NOS dimensions consequently take on discipline-specific meanings in geography through spatial and scalar reasoning, diverse and mediated evidence, methodological plurality, modelling, human-environment synthesis, and socially situated decision-making. FRA provides a common analytical structure for textbook analysis, while these disciplinary characteristics guide the interpretation of its dimensions in geographical contexts.

### 2.4. NOS, geography textbooks, and education for sustainable development

The disciplinary features described above take on added relevance in Education for Sustainable Development (ESD). Issues such as climate change, resource use, environmental degradation, urban development, disaster risk, and human-environment coordination involve intertwined natural and human processes. Many have the characteristics of “wicked problems,” with uncertainty or disagreement surrounding problem definitions, relevant knowledge, stakeholder priorities, and possible responses [8]. Geography is closely aligned with ESD because it brings human-environment relationships, spatial differentiation, regional context, and multiple scales into the analysis of sustainability problems [4].

Sustainability places epistemic demands on geographical education that go beyond the acquisition of established facts or the identification of technically feasible solutions. These issues often combine questions of knowledge with ethical and political challenges [9]. Evidence may be incomplete, uncertain, or open to different interpretations; methods and models may emphasize different aspects of the same problem; and conclusions reached at one scale or in one region may not transfer directly to another. Decisions can also involve judgments about priorities, responsibilities, risks, acceptable trade-offs, and the distribution of costs and benefits. Understanding sustainability-related geographical issues therefore requires attention to how evidence is generated and evaluated, how methodological and modelling choices shape explanations, what uncertainties accompany knowledge claims, and the conditions under which those claims remain applicable.

The social context of sustainability adds another reason for considering NOS. Decisions about environmental protection, resource exploitation, urban development, disaster governance, land-use change, or regional planning are seldom settled by evidence alone. Scientific information enters settings in which institutions, communities, stakeholders, and policy actors may carry different responsibilities, interests, and value commitments. Knowledge then becomes part of negotiation, priority setting, and collective action. Sustainability-related geographical reasoning therefore depends on understanding how evidence and methods interact with values, institutions, and action, especially where scientific knowledge informs but does not determine a single solution.

This concern maps closely onto the two-system structure of FRA. The cognitive-epistemic dimensions address scientific knowledge, practices, methods and methodological rules, and epistemic aims and values; the social-institutional dimensions address the norms, social values, organizations, interactions, professional structures, and power relations involved in producing and using scientific knowledge [10]. The two systems can be distinguished analytically in sustainability contexts, although in practice they are intertwined. Evidence and methods contribute to problem definition and explanation, while the evaluation and implementation of responses may also depend on institutional arrangements, public priorities, social values, political processes, and negotiation among actors. Making these relationships explicit is one way in which NOS becomes relevant to ESD.

Geography textbooks provide one curricular setting in which these relationships can appear. The presence of sustainability topics, however, does not by itself show whether their epistemic and social complexity is made explicit. Nguyen [40] found that ESD content in Vietnamese geography textbooks was presented mainly through descriptive and transmissive approaches. Such treatment can communicate sustainability knowledge while leaving the investigation of problems, the evaluation of competing explanations, and the connection between evidence, choice, and action relatively implicit. Biström and Lundström [41] reported a similar limitation in Swedish lower-secondary geography and biology textbooks, where sustainable-development content offered limited support for the multidimensional and conflict-oriented reasoning associated with action competence. These studies point to a distinction between the presence of sustainability content and the way textbooks represent the knowledge-making and decision-making involved in addressing sustainability issues.

For geography textbooks, this distinction matters because sustainability content can be extensive while the underlying knowledge-making processes remain only partly represented. A textbook may explain environmental degradation, introduce resource-conservation technologies, or describe regional-development policies without giving comparable attention to uncertainty in the evidence, assumptions built into models, the roles of scientific or professional communities, differences among stakeholder interests, institutional negotiation, or conflicts among values associated with alternative actions. Under such conditions, links between the cognitive-epistemic and social-institutional dimensions remain weak even though sustainability topics are present. NOS analysis provides a way to examine the conception of knowledge and problem solving embedded in the treatment of sustainability-related geographical issues.

The coding analysis remains grounded in the established FRA dimensions. Sustainable development is used as an interpretive context for considering why the observed distribution and relational organization of those dimensions matter for geography education. The main analytical concern is the way textbook representations of geographical knowledge connect knowledge, evidence, methods, practices, representations, values, institutions, and action. The presence of sustainability-related topics is considered within this broader pattern, because these relationships become especially consequential in sustainability contexts.

A more specific gap emerges where NOS research, geography textbook research, and ESD intersect. NOS textbook studies have focused mainly on conventional science subjects, while studies of sustainability in geography textbooks have more often examined the presence, organization, or pedagogical treatment of ESD content. Much less is known about how geography textbooks connect the cognitive-epistemic and social-institutional dimensions of NOS, especially in curriculum contexts where geographical knowledge is expected to inform the understanding of complex human-environment problems. Examining the distribution of FRA dimensions together with their relational patterns can provide evidence about the textual opportunities and limitations through which geography textbooks represent the nature of geographical knowledge in the context of sustainable development.

## 3. Theoretical analytical framework

**Fig 1:**
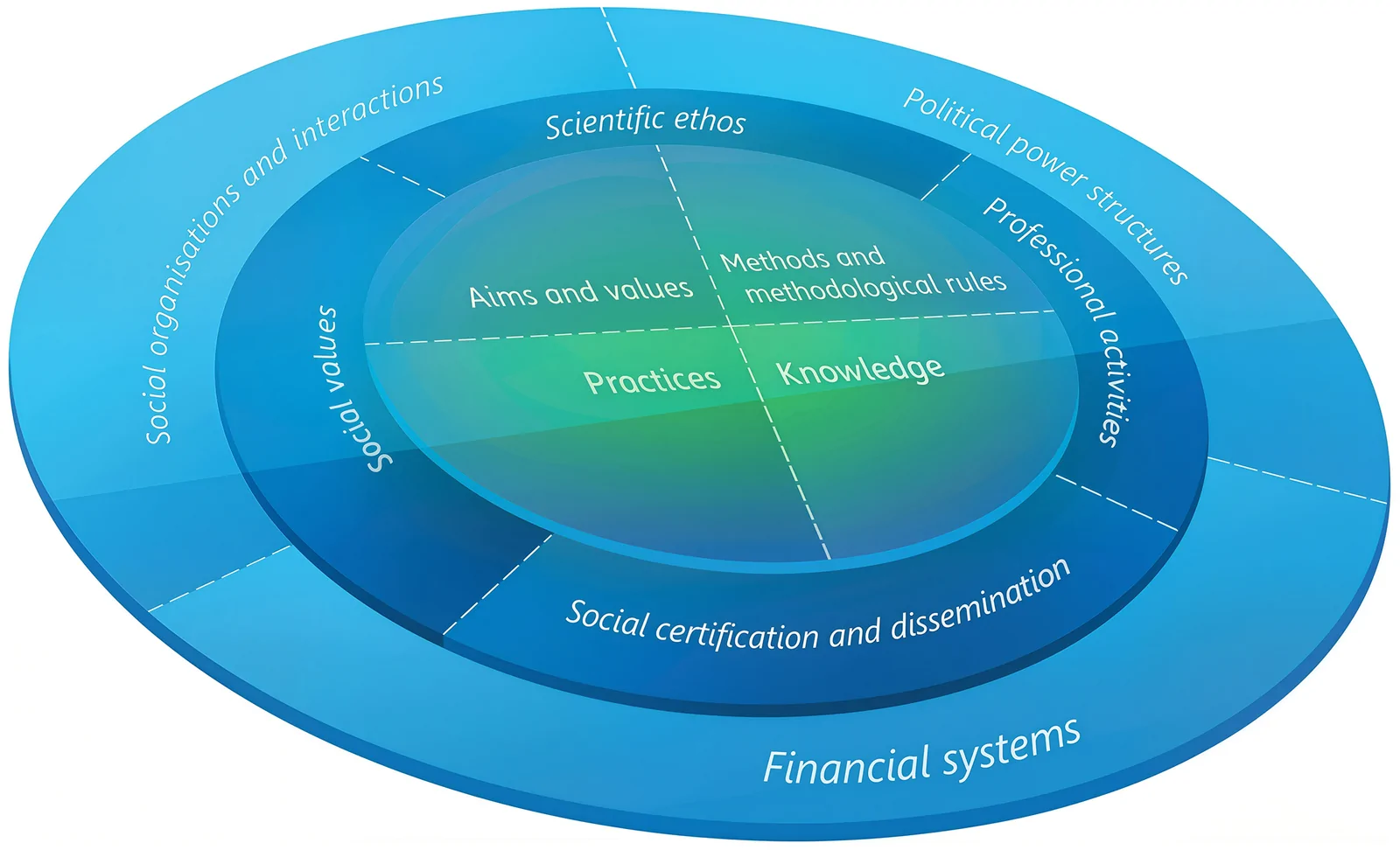
The FRA wheel: science as a cognitive-epistemic and social-institutional system [10].

This study adopts the Family Resemblance Approach (FRA) proposed by Erduran and Dagher [10] as its theoretical foundation for analyzing NOS representation in geography textbooks. The FRA framework identifies distinct dimensions or ‘clusters of characteristics’ to compare and describe different disciplines, portraying the nature of science as a holistic system [10]. The FRA Wheel conceptualizes NOS as a system encompassing two interrelated domains—the cognitive-epistemic domain and the social-institutional domain—which together consist of eleven interrelated categories. Specifically, the cognitive-epistemic domain includes aims and values, methods and methodological rules, scientific practices, and scientific knowledge. The social-institutional domain encompasses professional activities, scientific ethos, social values, social certification and dissemination, social organization and interaction, economic systems, and political power structures. Fig 1 presents the FRA Wheel visualization, with the innermost circle representing cognitive-epistemic elements and the outer circle illustrating social-institutional components. These elements are interconnected and mutually influential, reflecting the dynamic and integrated nature of science.

Previous FRA-based studies of NOS in textbooks, including recent work on Chinese high school physics textbooks [42], provide a basis for operationalizing the framework for textbook discourse analysis. The present study preserves the theoretical boundaries of FRA while translating several dimensions into coding categories that can be observed and applied consistently in textbook text. The distinction between the cognitive-epistemic and social-institutional systems is retained, and several categories are clarified at the coding level to improve transparency and consistency.

In the original FRA framework, “scientific ethos” refers to the normative commitments and value orientations that guide scientific communities and scientific work. In textbook discourse, however, these commitments are usually represented through concrete norms of scientific inquiry and scientific conduct, such as openness, honesty, skepticism, respect for evidence, and responsibility toward society and the environment. Therefore, this study used “Scientific Norms” as an operational coding label to identify textbook representations of scientific ethos.

The original FRA framework also distinguishes economic systems and political power structures as social-institutional dimensions. In the geography textbooks analyzed in this study, these two dimensions often appear in an intertwined manner, especially in content related to national policy, resource allocation, environmental governance, technological application, regional development, and sustainability planning. To improve coding consistency and avoid artificially separating closely related textbook content, this study combined these two dimensions into “Social Power Structures.” This category captures how political, economic, and institutional forces shape the production, application, and dissemination of scientific knowledge in geography-related contexts.

In addition, the original FRA category of social certification and dissemination was not treated as an independent coding dimension in this study. In the analyzed geography textbooks, relevant content was usually embedded in descriptions of professional activities, institutional publication channels, or organizational dissemination of scientific information. Therefore, such content was coded under Professional Activities or Social Organization and Interaction according to its textual function. This decision helped avoid overlapping codes and improved the consistency of textbook coding.

Based on these considerations, this study developed an operationalized nine-dimensional FRA-based analytical framework, including four cognitive-epistemic dimensions and five social-institutional dimensions. This operationalized framework maintains the core logic of FRA while making the categories more suitable for systematic textbook coding and ENA analysis.

For coding convenience, each dimension was assigned an English abbreviation: cognitive–epistemic dimensions were prefixed with “C” (C.A.V: Cognitive Aims and Values; C.SP: Scientific Practice; C.M.MR: Methods and Methodological Rules; C.SK: Scientific Knowledge), and social–institutional dimensions were prefixed with “S” (S.PA: Professional Activities; S.SN: Scientific Norms; S.SV: Social values; S.SO.I: Social Organization and Interaction; S.PS: Social Power Structures).

The operationalized coding framework is presented in Table 1.

**Table 1:** Content structure of the nine-dimensional FRA-based framework for NOS.

Dimensions of the Nature of Science	Categories	Connotations	Operational Definition
Cognitive-epistemic aspects	Aims and values (C.A.V)	Scientific activity is underpinned by a coherent system of values that guide scientific practices and norms, although these values are often not explicitly stated.	This category includes epistemic aims and values that guide scientific inquiry and reasoning, such as accuracy, objectivity, consistency, skepticism, rationality, simplicity, empirical adequacy, predictability, testability, novelty, effectiveness, tentativeness, causality, logical coherence, feasibility, and explanatory power.
	Scientific Practice (C.SP)	Scientific activities encompass a broad range of cognitive, epistemic, and discursive practices. They include concrete practices such as observation and experimentation, as well as the use of specialized working techniques by scientists to support these practices. Additionally, scientific activities involve documenting procedural steps or research findings in various forms of records and reports.	This category includes scientific practices such as observation, experimentation, comparison, classification, modeling, specialized techniques, the use of geographic information technologies, digital technologies, preparation and planning, data recording, drawing, photography, and chart construction.
	Methods and methodological rules (C.M.MR)	Scientists conduct rigorous research through a variety of observational, investigative, and analytical methods to generate reliable evidence and construct disciplinary theories, laws, and models. These methods are guided by specific methodological rules and may be modified according to research purposes, evidence types, and disciplinary contexts. Different methods produce different forms of evidence, thereby deepening the understanding of phenomena and providing more coherent explanations.	This category includes specific scientific investigation methods, analytical procedures, methodological rules, and explanations of how scientific methods are selected, modified, or justified.
	Scientific knowledge (C.SK)	Theories, laws, and models are interrelated outcomes of scientific activities. They are used to generate, organize, and justify scientific knowledge and to provide logically consistent explanations of phenomena. Scientific knowledge is holistic and interrelated, and should be regarded as a coherent network rather than as discrete and disconnected fragments of knowledge.	This category includes geographical concepts, scientific hypotheses, theories, models, principles, laws, and other forms of established scientific knowledge.
Social-institutional aspects	Professional Activities (S.PA)	Scientists engage in a variety of professional activities related to producing, evaluating, communicating, and sustaining scientific work, including attending and organizing academic conferences, publishing peer-reviewed articles, reviewing manuscripts, and applying for research funding.	This category includes scientific historical accounts, biographical introductions, or descriptions of scientific work that involve professional activities, such as publishing research findings, participating in scientific meetings, applying for research support, and receiving honors or awards.
	Scientific Norms (S.SN)	Scientists are expected to follow a set of norms in their own research and in their interactions with other members of the scientific community. These norms may include organized skepticism, universalism, communalism, disinterestedness, openness, intellectual honesty, respect for research subjects, and responsibility toward the environment.	This category includes ethical and normative requirements of scientific work, such as respect for research subjects, environmental responsibility, protection of human participants, confidentiality, openness, sharing, and legal compliance.
	Social values (S.SV)	The scientific enterprise serves societal development by addressing issues such as environmental protection, sustainable development, and responding to human needs.	This category includes the societal value of geography or scientific knowledge, such as contributions to sustainable development, environmental conservation, public well-being, technological progress, industrial production, and social development.
	Social Organization and Interaction (S.SO.I)	Science is organized through institutions such as universities and research centers. Social interactions within research teams are influenced by organizational hierarchies; at a broader level, scientific institutions often maintain connections with industry and defense sectors.	This category includes teamwork, scientific collaboration, research institutions, organizational structures, and interactions among scientists, institutions, government agencies, industry, and other social actors.
	Social Power Structures (S.PS)	Scientific activities take place within political contexts and are mediated by economic factors. These environments impose their own values and interests while constraining scientific work. Researchers rely on funding support to conduct their work, which in turn influences research topics, types of sponsorship, and the nature of scientific activities. Science is not universally neutral or unimpeded; its outcomes do not necessarily benefit all individuals, groups, or cultures equally.	This category includes the influence of political, economic, and institutional power structures on scientific research, the application of scientific knowledge, resource allocation, environmental governance, industrial development, commercialization, and research funding.

## 4. Materials and methods

Epistemic Network Analysis (ENA) is a network-based analytical method designed to examine and visualize relationships among elements through their co-occurrence patterns within defined analytical units. Rather than focusing only on the frequency of individual codes, ENA captures the structure and strength of co-occurrences, thereby revealing dynamic interactions among elements within a cognitive, social, and interactional system [43]. This focus on relational patterns allows for a more meaningful interpretation of how different dimensions of the Nature of Science (NOS) are interconnected.

Given the broad conceptual scope of the Family Resemblance Approach (FRA), ENA is particularly suitable for illustrating interactions between the cognitive-epistemic system and the social-institutional system of science. By mapping these relationships as networks, ENA extends the analytical potential of FRA, enabling researchers to examine how multiple NOS dimensions jointly operate within curriculum materials. This approach has been applied in science education research to analyze curriculum content and assessments coded under the FRA framework [26]. In the present study, the two broad FRA domains and nine NOS dimensions were integrated to examine co-occurrence patterns across the selected textbooks.

The selected sample consisted of four Chinese senior secondary compulsory geography textbooks published by People’s Education Press and Hunan Education Press. The four textbooks were PEP Geography Compulsory Book 1 (人教版普通高中教科书·地理·必修 第一册, People’s Education Press, 2019 edition), PEP Geography Compulsory Book 2 (人教版普通高中教科书·地理·必修 第二册, People’s Education Press, 2019 edition), HNEP Geography Compulsory Book 1 (湘教版普通高中教科书·地理·必修 第一册, Hunan Education Press, 2019 edition), and HNEP Geography Compulsory Book 2 (湘教版普通高中教科书·地理·必修 第二册, Hunan Education Press, 2019 edition). In the subsequent analysis, the four textbooks are consistently abbreviated as PEP1, PEP2, HNEP1, and HNEP2. All coding lines were binary-coded according to the nine-dimensional FRA-based framework operationalized in this study. Each NOS dimension was coded as “1” when it was represented in a coding line and “0” when it was not represented. A single coding line could contain more than one NOS dimension.

These four textbooks were selected because they are mainstream Chinese senior secondary compulsory geography textbooks developed under the national geography curriculum standards and published by two major textbook publishers in China. The inclusion of both Compulsory Book 1 and Compulsory Book 2 enables the study to cover physical geography and human geography content, which are the two major components of the compulsory senior secondary geography curriculum. Therefore, the sample is appropriate for examining the representation of NOS in mainstream Chinese senior secondary compulsory geography textbooks.

However, the study does not claim that these four textbooks fully represent all geography textbooks used in China across all grades, publishers, and curriculum modules. The findings should be interpreted within the specific scope of Chinese senior secondary compulsory geography textbooks represented by the selected textbook versions. Future studies may include additional textbook versions, elective modules, and international textbook samples to further examine the generalizability of the findings.

Epistemic Network Analysis (ENA) was used to examine co-occurrence relationships among the nine NOS dimensions. The coded dataset was analyzed using the ENA Web Tool (https://app.epistemicnetwork.org). The textbook-version variable was used as both the unit of analysis and the horizon, yielding four units corresponding to PEP1, PEP2, HNEP1, and HNEP2 and preventing moving windows from crossing textbook boundaries. The nine FRA-based NOS dimensions—C.A.V, C.SP, C.M.MR, C.SK, S.PA, S.SN, S.SV, S.SO.I, and S.PS—were entered as binary codes.

A standard ENA model with a four-line moving window was used. Because a complete sentence generally served as one coding line, the window moved through the coding lines in their original textual order, and co-occurrences among NOS dimensions were accumulated within each textbook. A Standard Network was used to represent undirected co-occurrence relationships among NOS dimensions within the defined textual window. No additional weighting was applied. The accumulated co-occurrence vectors were sphere-normalized before dimensional reduction using singular value decomposition (SVD).

In the ENA network figures, each node represents one NOS dimension, and each edge represents a co-occurrence relationship between two dimensions. Edge thickness reflects the relative strength of the corresponding co-occurrence relationship, with thicker edges indicating stronger connections.

The coding process was conducted in several stages. First, two coders received systematic training on the Family Resemblance Approach (FRA) framework and the nine-dimensional NOS coding scheme used in this study. The training focused on the conceptual meanings, operational definitions, and coding examples of each NOS dimension. During the training stage, selected textbook excerpts were used as training samples to help the coders establish a shared understanding of the coding criteria.

Second, a pilot coding stage was conducted. The two coders independently coded selected textbook samples and then compared their coding results. Discrepant cases were discussed in relation to the operational definitions of the coding framework. This process helped refine the coding criteria and reduce ambiguity before formal coding.

Third, formal coding was conducted. A complete sentence was generally treated as one coding line. Each coding line was coded according to the nine NOS dimensions, with “1” indicating the presence of a given NOS dimension and “0” indicating its absence. A single coding line could include more than one NOS dimension. A total of 3,942 coding lines were included in the formal coding. To assess inter-coder reliability, a 25% subset of all coding units was independently coded by the two coders, resulting in a reliability sample of 986 coding lines. The sample was selected using a stratified and systematic sampling procedure. Specifically, textbook version was used as the stratification variable, and within each stratum, sentences were sampled at fixed intervals according to their original order in the textbook text. This procedure ensured that the sampled units were evenly distributed across chapters and covered the full text, thereby avoiding over-representation of any particular section. Based on the two coders’ independent coding results before discussion, the sentence-level exact agreement was 83%, and the pooled Cohen’s kappa across the nine binary NOS categories was 0.66, indicating an acceptable level of inter-coder reliability.

Finally, all disagreements identified during the reliability check were reviewed and discussed by the two coders. Cases that remained difficult to judge were further examined with reference to the operational definitions of the coding framework. After discussion, consensus was reached on all NOS-related coding decisions. The finalized coded dataset was then used for frequency analysis and Epistemic Network Analysis.

Chinese senior secondary compulsory geography textbooks include diverse content forms such as main text, sidebars, extension sections, images, and exercises. To ensure analytical consistency and comparability, all of these content types were included in the coding process. An example of the ENA coding procedure is provided in Table 2.

**Table 2:** Example of ENA coding.

Textbook Version	Chapter Theme	Section Title	Specific Content	C.A.V	C.SP	C.M.MR	C.SK	S.PA	S.SN	S.SV	S.SO.I	S.PS
HNEP2	Chapter 4: Regional Development Strategy	Section 3: Maritime Rights and Interests and China’s Maritime Development Strategy	China should follow a strategy of science and technology first, vigorously develop marine high technology, strive to break through technical bottlenecks that constrain marine economic development and ecological protection, and occupy the commanding heights in implementing the maritime strategy.	0	1	0	0	0	0	1	0	1

## 5. Results

**Fig 2:**
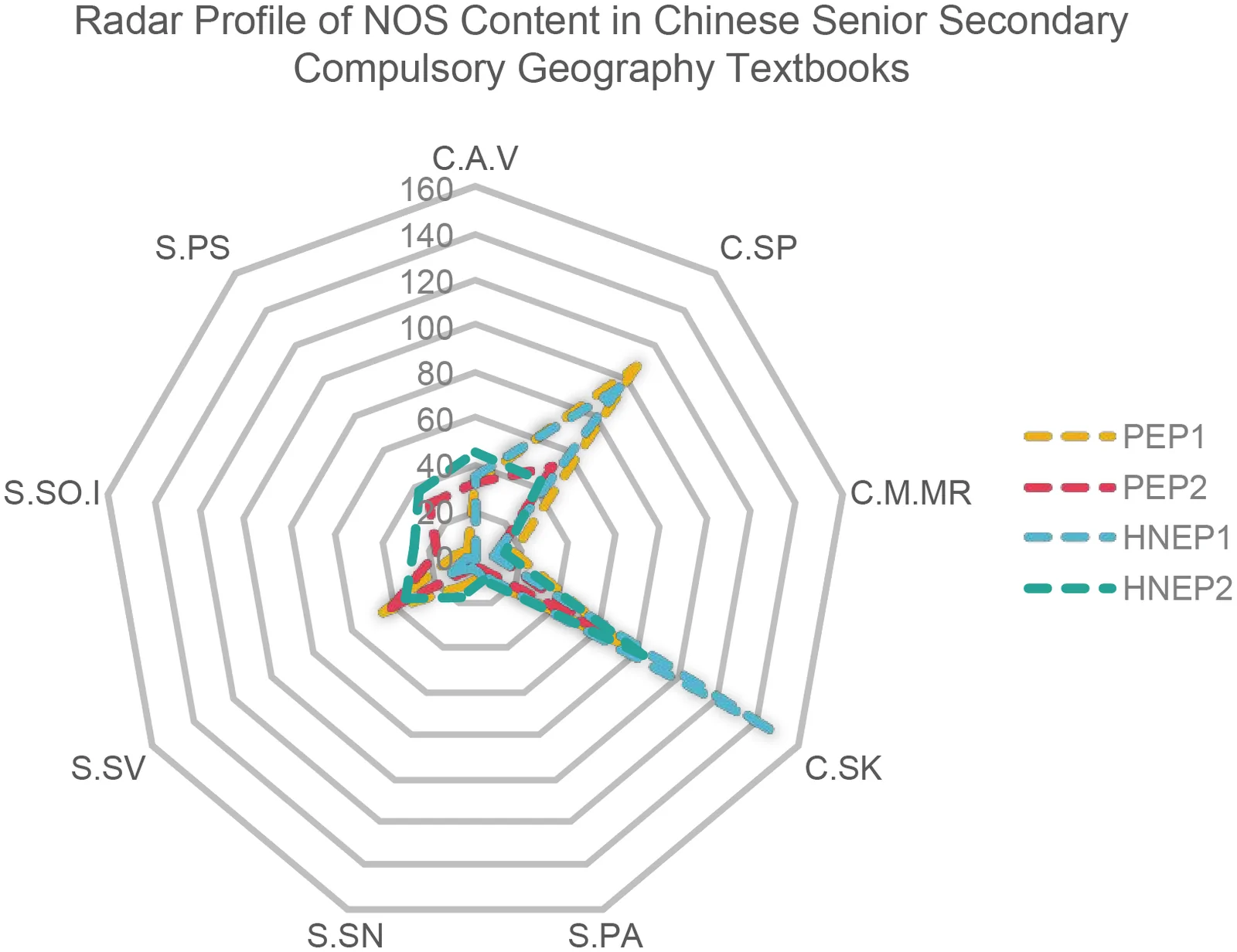
Comparative radar profiles of the nine NOS dimensions across PEP1, PEP2, HNEP1, and HNEP2.

Fig 2 presents the comparative radar profiles of the nine NOS dimensions across the four selected textbooks: PEP1, PEP2, HNEP1, and HNEP2.

The radar profiles indicate that the most prominent dimensions are Scientific Knowledge (C.SK) and Scientific Practice (C.SP), suggesting that the textbooks place strong emphasis on geographical concepts, principles, and practice-oriented learning activities. By contrast, the social-institutional dimensions, including Professional Activities (S.PA), Scientific Norms (S.SN), and Social Organization and Interaction (S.SO.I), appear at relatively low frequencies. Social Power Structures (S.PS) are also unevenly represented across the four textbooks, indicating comparatively limited textual representation of how geographical issues intersect with governance, environmental planning, resource allocation, and land-use decision-making. The relatively low frequency of Cognitive Aims and Values (C.A.V) further indicates that value-oriented aspects of scientific inquiry, such as accuracy, critical skepticism, and rational judgment, are less explicitly foregrounded in the textbook discourse.

### 5.1. Overall distribution of NOS representations in the four textbooks

Overall, a total of 1,196 instances of Nature of Science (NOS) representations were identified across the four Chinese senior secondary compulsory geography textbooks: PEP1, PEP2, HNEP1, and HNEP2. Among these, 863 instances (72.2%) were related to the cognitive-epistemic system, while 333 instances (27.8%) belonged to the social-institutional system. Across the sample as a whole, the nine NOS dimensions defined within the Family Resemblance Approach (FRA) were represented, but not every dimension appeared in each textbook, and the frequency of representation varied markedly across categories. This variation is partly attributable to the thematic orientation of the textbooks: Compulsory Book 1 in both textbook series focuses mainly on physical geography, whereas Compulsory Book 2 emphasizes human geography, leading to notable differences in the distribution and emphasis of NOS elements.

The frequency distribution of NOS dimensions is shown in Table 3, and the proportions of the two FRA systems are summarized in Table 4.

**Table 3:** Frequencies of NOS dimensions across the four textbooks.

Textbook version	C.A.V	C.SP	C.M.MR	C.SK	S.PA	S.SN	S.SV	S.SO.I	S.PS
PEP1	33	109	17	74	8	12	46	9	5
PEP2	33	52	9	61	4	4	42	17	31
HNEP1	36	97	8	147	7	4	12	4	0
HNEP2	46	44	13	84	10	18	35	27	38

**Table 4:** Proportions of cognitive-epistemic and social-institutional NOS representations across the four textbooks.

Textbook version	Cognitive-epistemic aggregate	Social-institutional aggregate	Cognitive-epistemic proportion	Social-institutional proportion
PEP1	233	80	74.4%	25.6%
PEP2	155	98	61.3%	38.7%
HNEP1	288	27	91.4%	8.6%
HNEP2	187	128	59.4%	40.6%

The aggregate difference between the two FRA systems is not distributed evenly across individual dimensions. The dimensional profile therefore provides a more informative view of which aspects of geographical knowledge-making account for the overall pattern.

**Fig 3:**
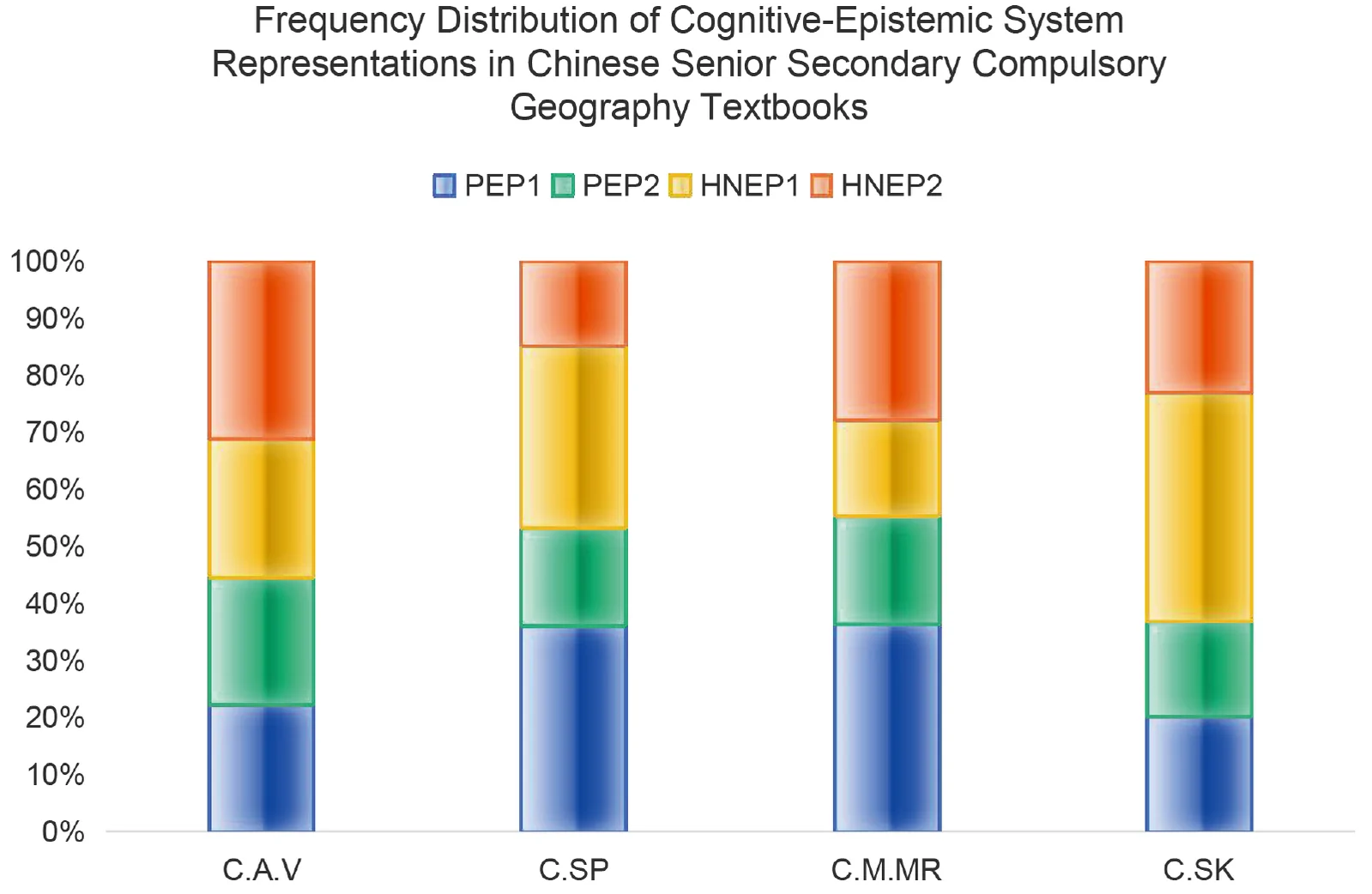
Relative distributions of cognitive-epistemic NOS dimensions across the four selected textbooks.

As shown in Fig 3, Scientific Knowledge and Scientific Practice constitute the largest proportions of cognitive-epistemic representations, followed by Cognitive Aims and Values, while Methods and Methodological Rules are the least represented. With the exception of PEP1, where Scientific Practice appears most frequently, Scientific Knowledge dominates in the remaining three textbooks. This pattern corresponds to the structure of PEP1, which emphasizes hands-on activities and their documentation, such as instructing learners to record the date, position, and shape of a crescent moon observed after sunset, together with the observation time. Such tasks make observation, recording, and practice-based reasoning explicitly visible as components of geographical knowledge-making.

Overall, the four textbooks foreground geographical principles and laws as central forms of disciplinary knowledge. Although Cognitive Aims and Values appear with relatively low frequency, some passages make processes of knowledge verification, evidence-based judgment, and scientific reasoning visible. Compared with the presentation of concepts, principles, and knowledge organization, however, Methods and Methodological Rules remain relatively limited. In particular, hypothesis testing, model construction, methodological justification, and the conditions under which methods are selected or modified receive comparatively little explicit treatment, identifying an important area for textbook improvement.

**Fig 4:**
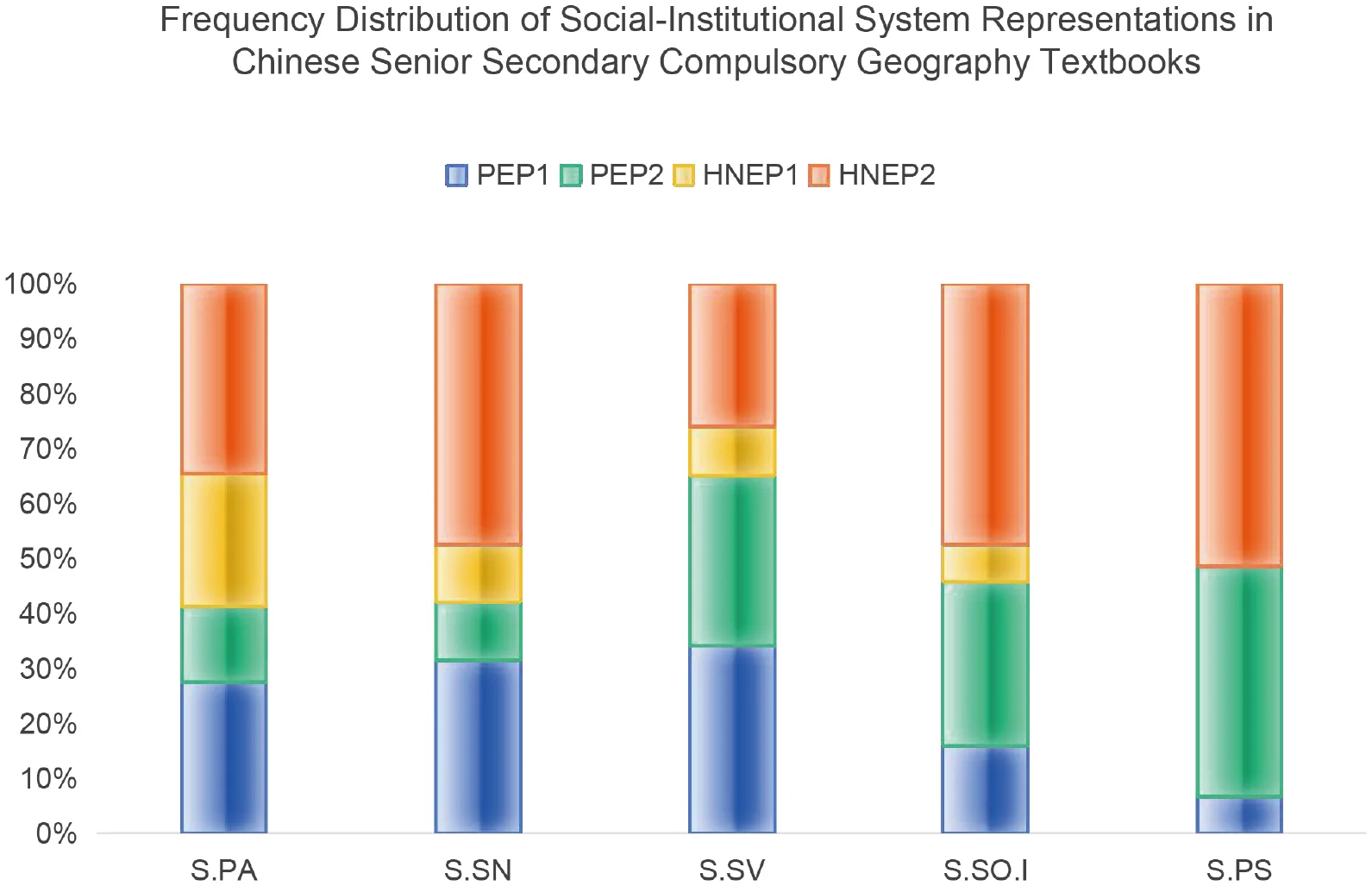
Relative distributions of social-institutional NOS dimensions across the four selected textbooks.

Fig 4 illustrates the distribution of social-institutional NOS representations across the four textbooks. Among these dimensions, Social values (S.SV) are the most frequently represented, indicating that the textbooks strongly emphasize the societal value and practical application of geographical knowledge. This emphasis reflects geography’s characteristic integration of natural processes and human systems, with human-environment coordination functioning as a central organizing principle of textbook content.

The second most prominent dimension is Social Power Structures (S.PS), which captures the influence of political, economic, and institutional contexts on scientific activity. Its representation varies markedly across the four textbooks, with no occurrences in HNEP1. This pattern suggests that textbooks with stronger human geography content provide more opportunities to present power-related NOS dimensions, whereas such dimensions are less visible in textbook content that focuses mainly on physical geography.

By contrast, Professional Activities, Scientific Norms, and Social Organization and Interaction appear infrequently. Where Social Organization and Interaction is addressed, it is largely limited to cooperation within single institutions or organizations, often illustrated through references to international organizations, such as the United Nations releasing scientific data or promoting green economy initiatives. Cross-institutional collaboration and the formation of broader scientific communities are rarely depicted.

Representations of Professional Activities are mainly confined to historical examples of individual scientific contributions, including Pierre Perrault’s publication of On the Origin of Springs (1674), Hu Huanyong’s identification of the Heihe–Tengchong Line, and Dokuchaev’s formulation of soil formation theory. These cases highlight scientific discovery but provide limited insight into the professional practices and institutional processes underlying knowledge production.

For Scientific Norms, the textbooks primarily present normative requirements embedded in scientific inquiry procedures, while ethical dimensions, such as respect for research subjects and the protection of human participants, are largely absent. Similarly, representations of Social Power Structures tend to illustrate linear links between science, policy support, and economic development, such as research investment enabling technological improvement, without examining the deeper embeddedness of scientific practice within political, economic, and institutional power relations.

The frequency profile shows a pattern of selective visibility. Established geographical knowledge, scientific practice, and the social usefulness of geography receive relatively explicit treatment, while professional practice, normative regulation, organizational interaction, and the institutional conditions of knowledge application receive less sustained attention. This pattern provides the basis for examining how the dimensions are connected in the ENA networks.

### 5.2. ENA network analysis of NOS representations in textbooks

**Fig 5:**
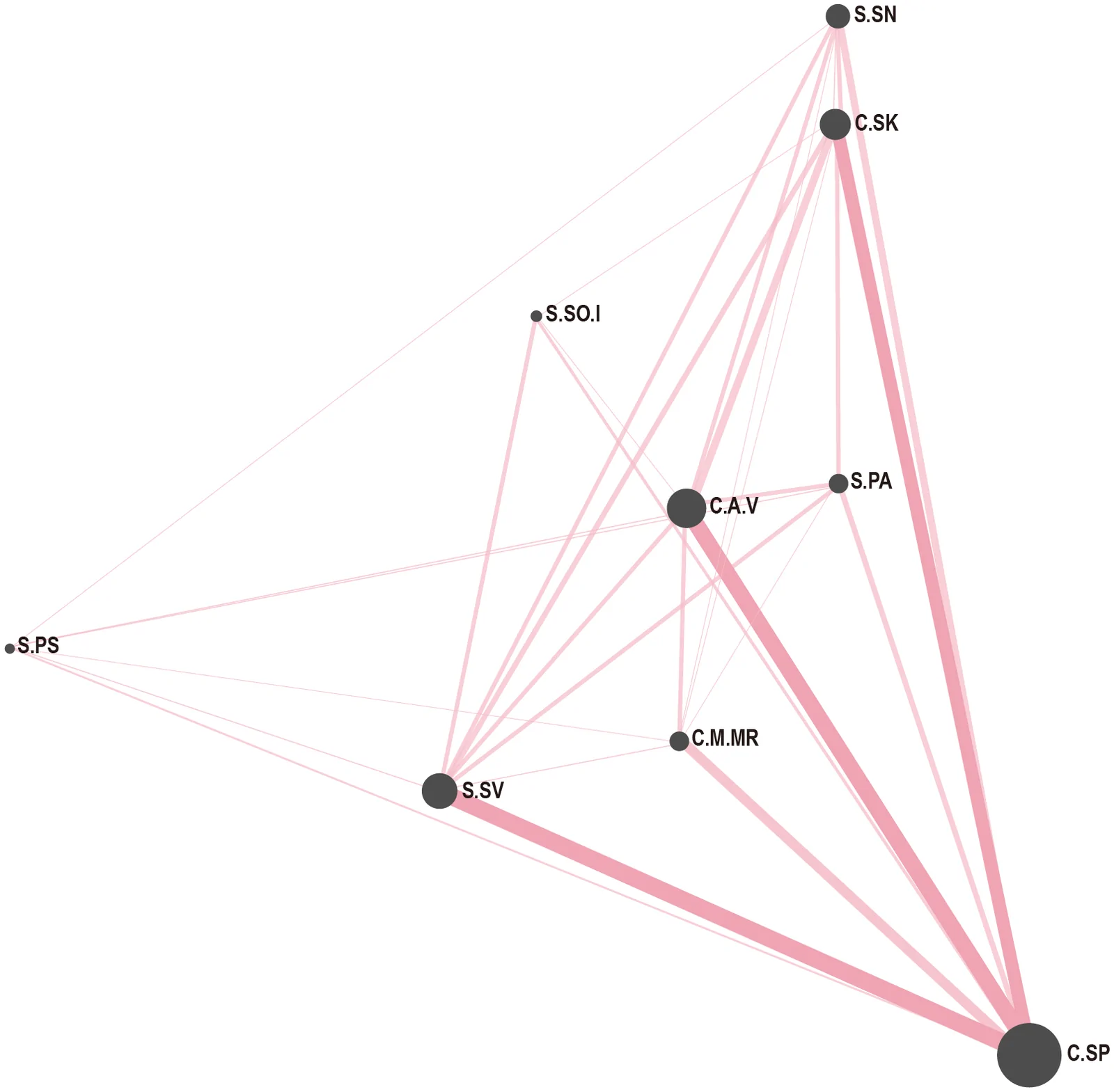
Overall NOS network in PEP1.

**Fig 6:**
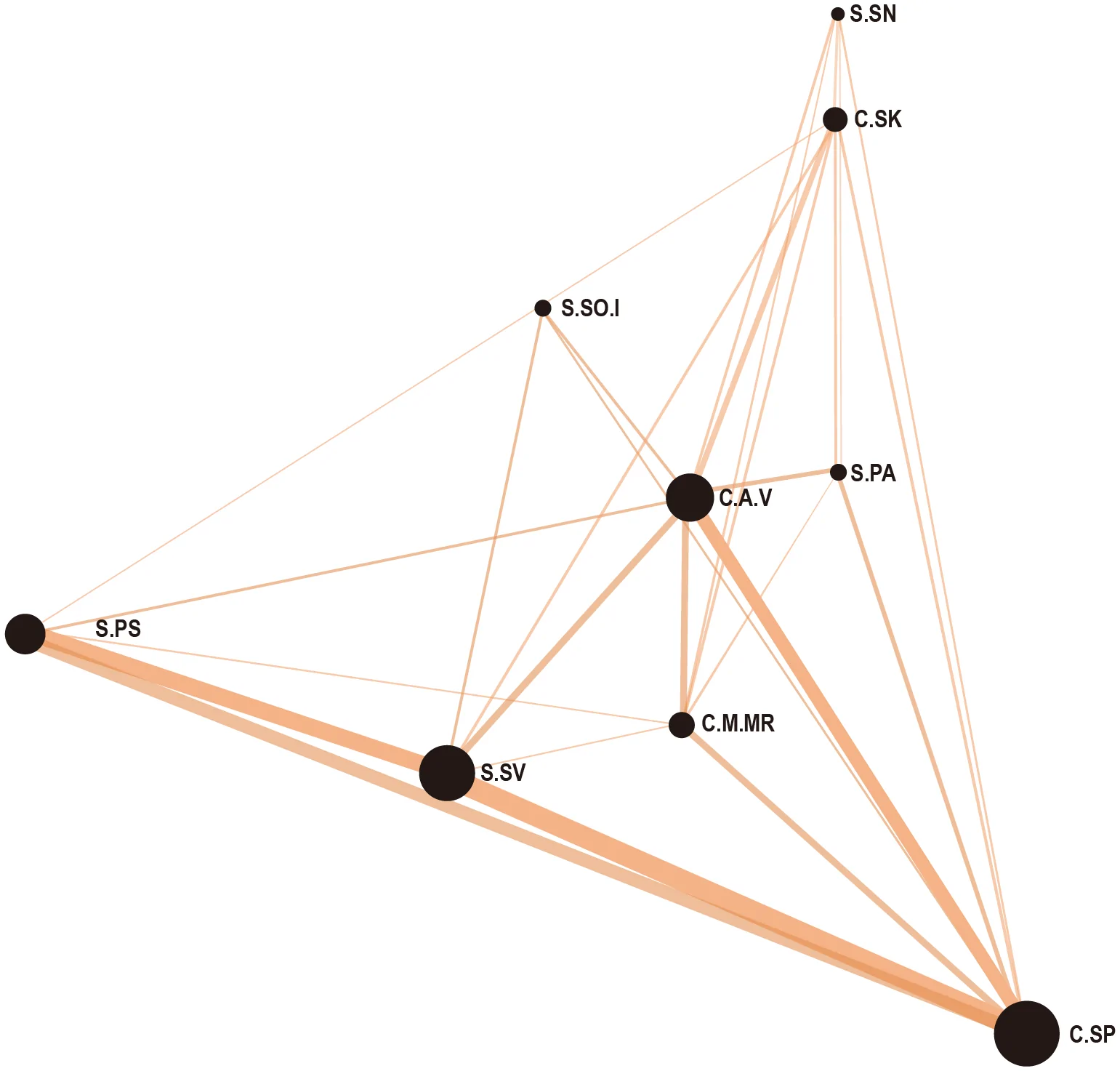
Overall NOS network in PEP2.

**Fig 7:**
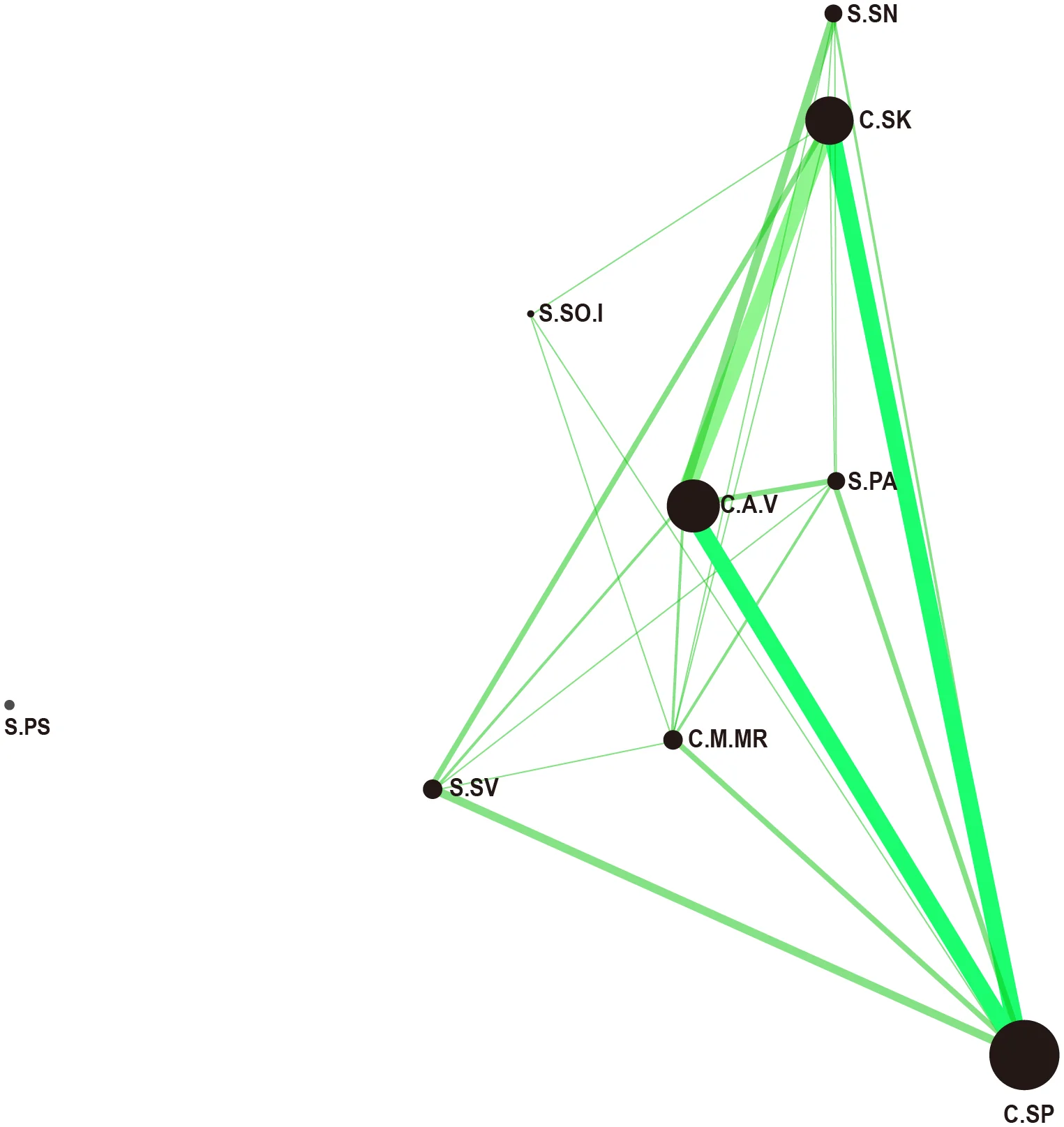
Overall NOS network in HNEP1.

**Fig 8:**
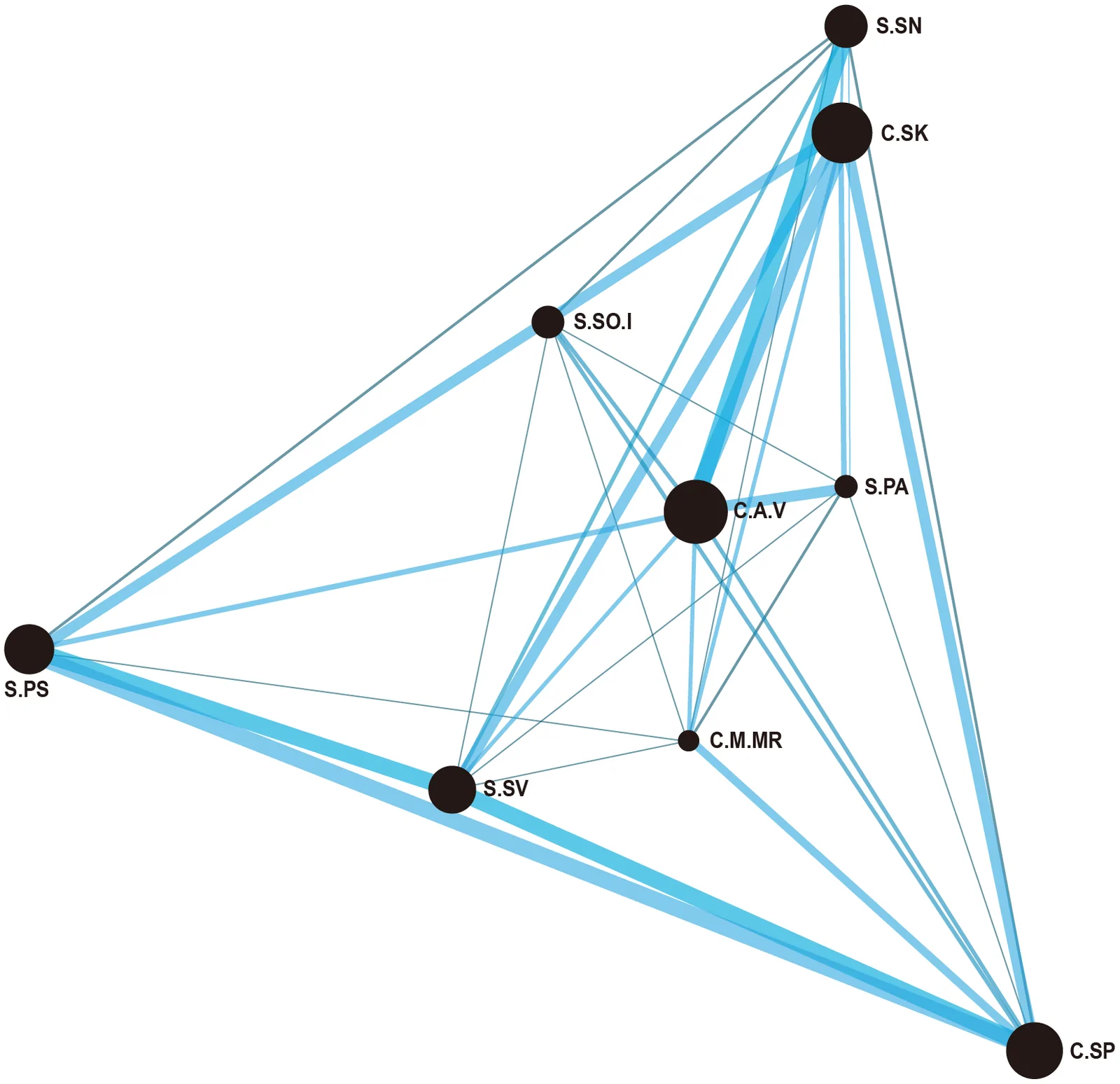
Overall NOS network in HNEP2.

The overall ENA networks for the four textbooks are presented in Figs 5–8. Each network diagram contains nine nodes representing the FRA-based NOS dimensions, and each edge represents the co-occurrence of two dimensions within the defined moving stanza window. The network analysis therefore shifts attention from how often individual categories appear to how they are organized within local sequences of textbook discourse.

#### Core structural patterns.

Across the four networks, the most stable configuration is the triangular relationship among Cognitive Aims and Values, Scientific Practice, and Scientific Knowledge. Methods and Methodological Rules form a secondary set of links with Scientific Practice and Scientific Knowledge, while Social values most often connect the social-institutional side of the framework to Scientific Practice. Professional Activities appear in fewer and narrower connections. These recurring configurations identify the principal relational architecture that is examined in the representative examples below.

A representative example of the recurrent C.A.V-C.SP-C.SK structure appears in PEP1 in the section on geological time. The textbook describes how scientists study strata and fossils to infer the history of life and paleo-geographical environments, compare strata and paleontological fossils from different regions, and use stratigraphic sequence, stages of biological evolution, rock ages, and other evidence to establish the geologic time scale. These passages connect epistemic aims and evidential reasoning with scientific practice and established geographical knowledge. Methodological reasoning is also present through the comparison and interpretation of different forms of geological evidence. This example illustrates how several cognitive-epistemic dimensions are integrated within the presentation of geographical knowledge rather than appearing as isolated NOS elements.

#### Substructural patterns.

Within the social-institutional system, Social values and Social Power Structures are connected in some networks through content concerning the social application of geographical knowledge and the influence of political or economic conditions. Professional Activities and Scientific Norms occur together only occasionally, as do Social Organization and Interaction and Social values. Rather than forming a comparable multi-node cluster, these categories tend to appear in narrower pairwise relations, usually around particular applications or institutional references.

A contrasting example from PEP2 shows how social-institutional dimensions are represented less fully even when geographical knowledge is placed in an applied context. In the section on urban management, the textbook describes the use of geographic information technology in urban planning, municipal construction, public services, and government and enterprise applications, including the use of GIS to optimize the layout of public-service facilities. The passage connects scientific practice with the social value and institutional application of geographical knowledge. However, it gives little attention to interaction among institutions or stakeholder groups, the negotiation of competing interests or values, or the norms that might guide such decisions. The example therefore illustrates how socially relevant applications of geographical knowledge can be made visible while several social-institutional relationships remain comparatively underrepresented.

**Fig 9:**
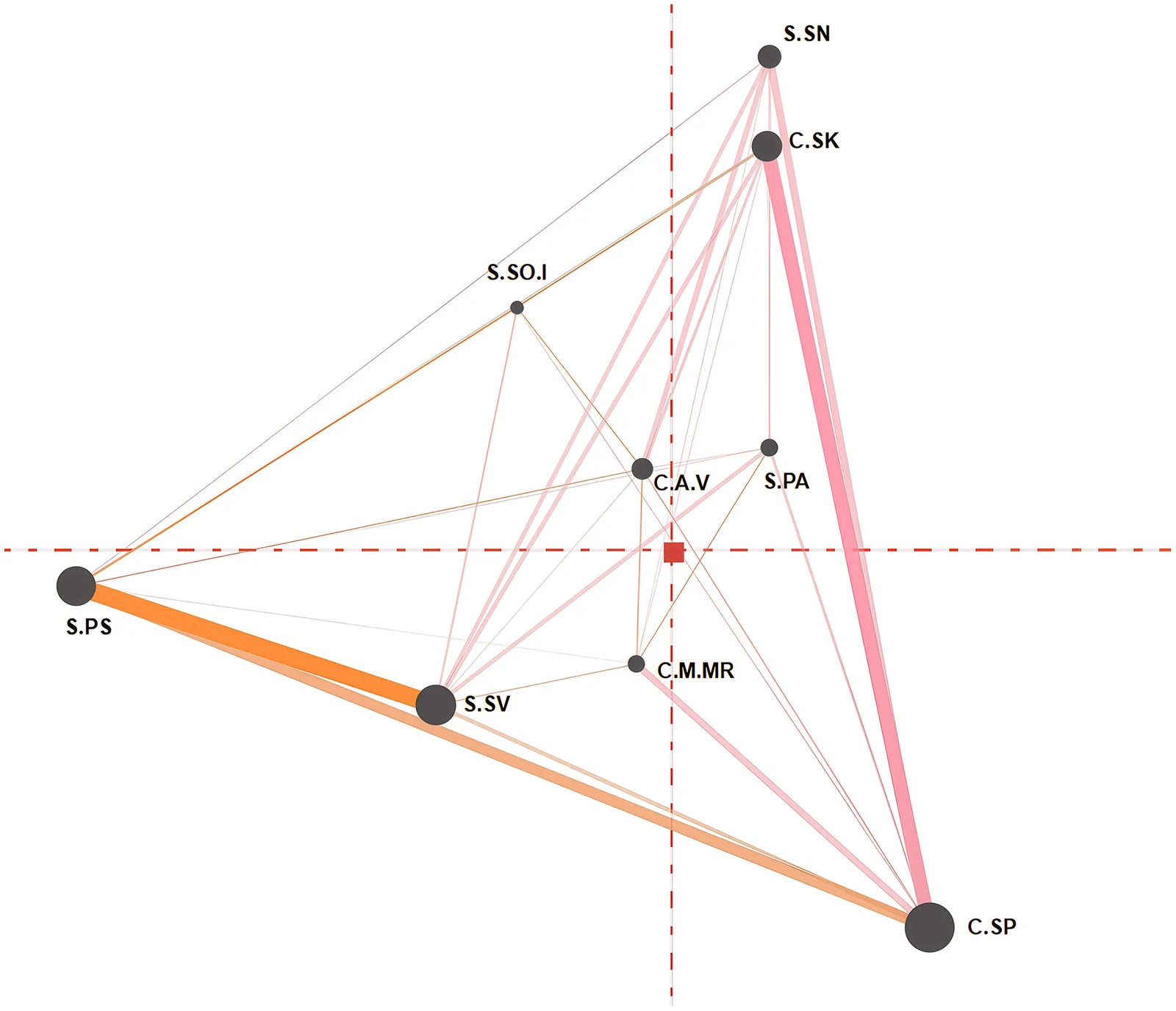
Difference network of NOS representations between PEP1 and PEP2.

**Fig 10:**
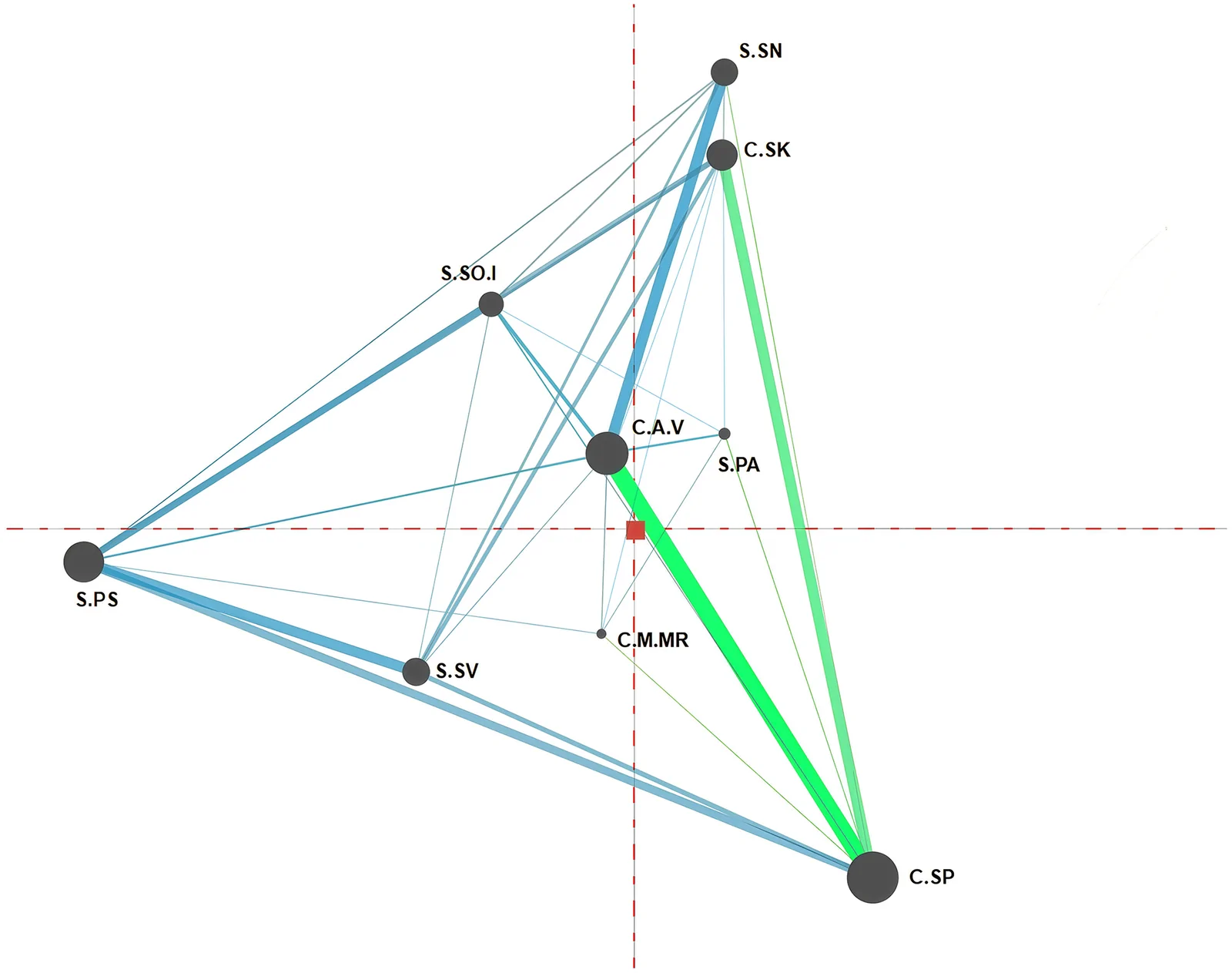
Difference network of NOS representations between HNEP1 and HNEP2.

**Fig 11:**
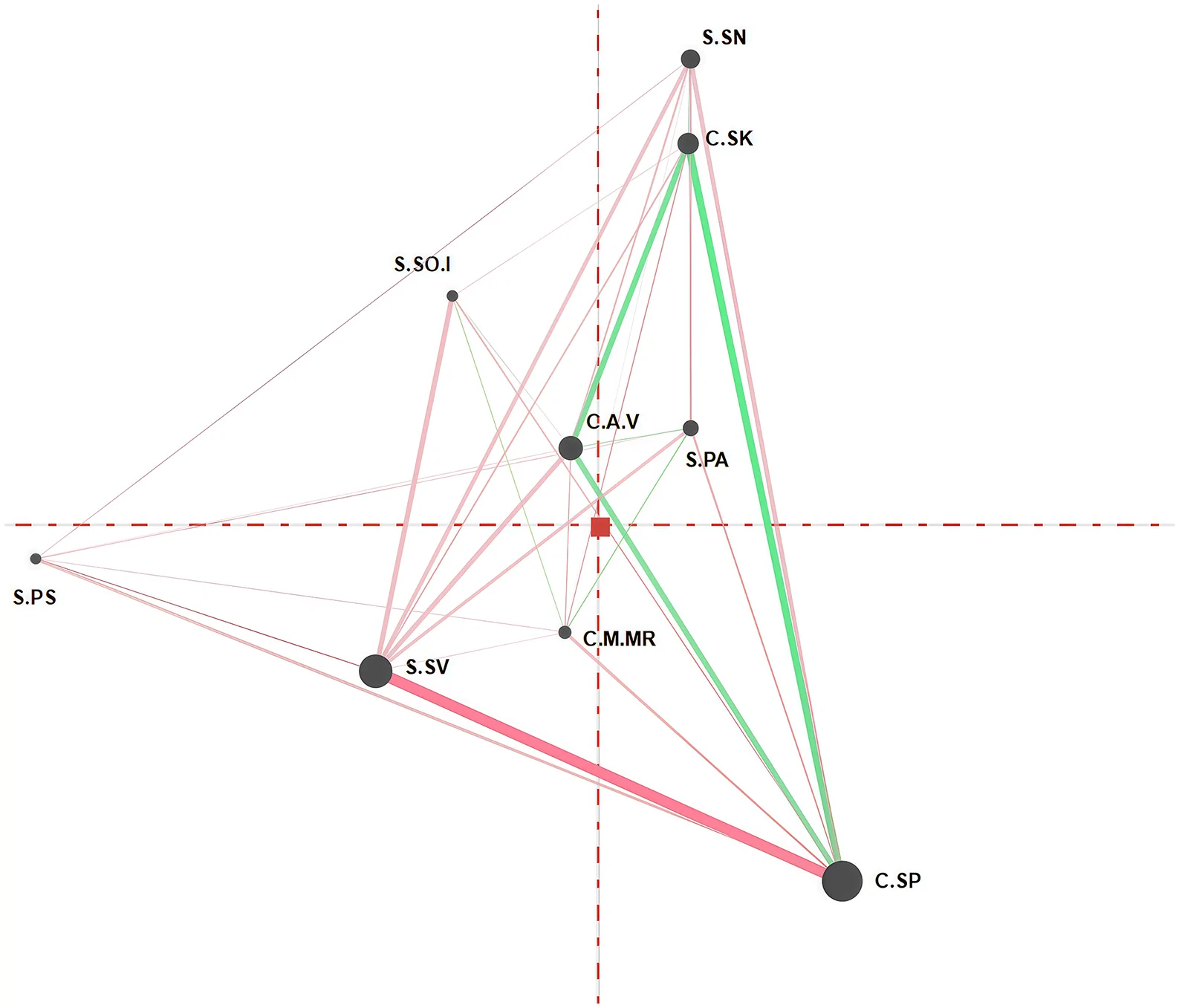
Difference network of NOS representations between PEP1 and HNEP1.

**Fig 12:**
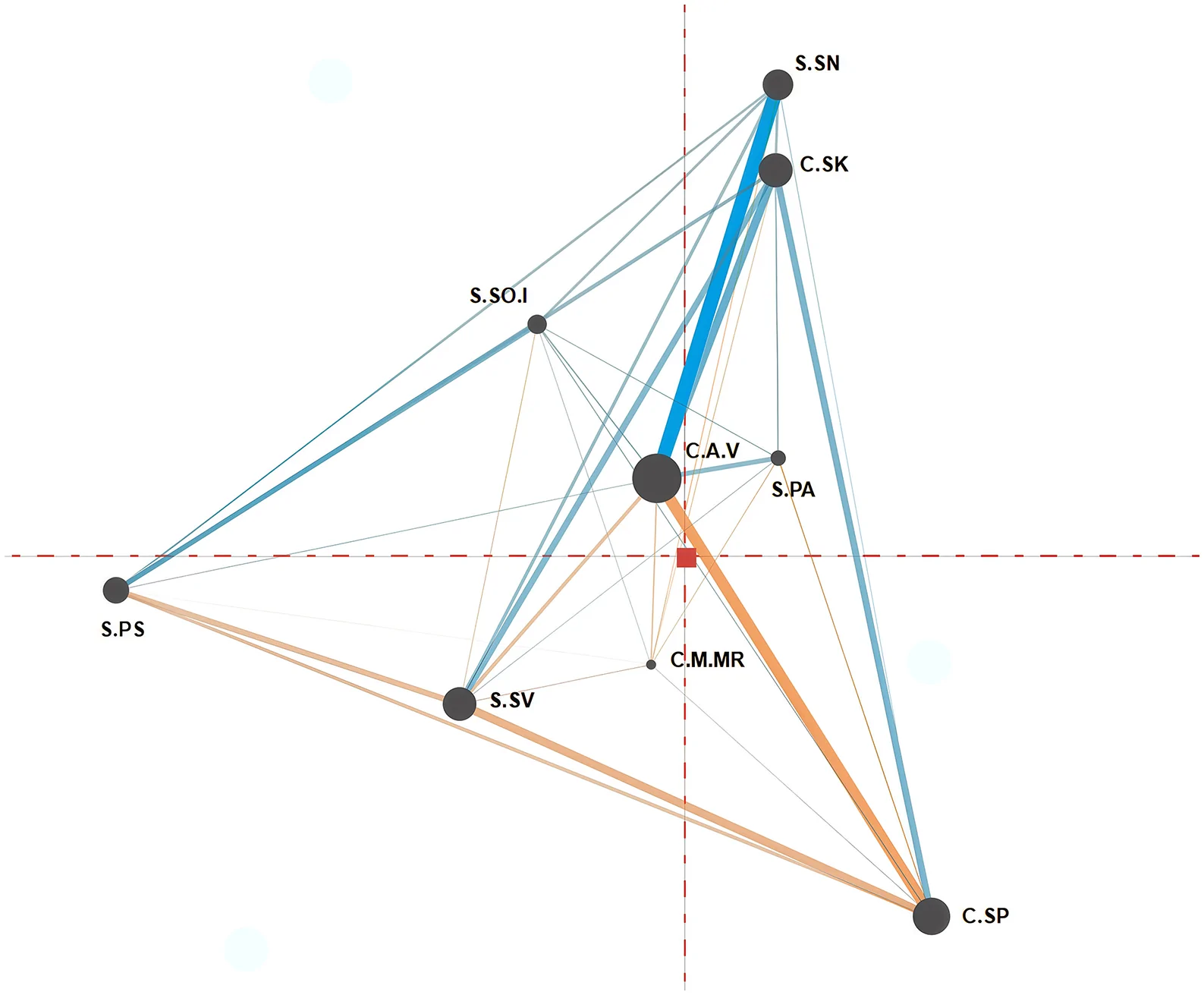
Difference network of NOS representations between PEP2 and HNEP2.

Difference networks were generated by comparing the edge weights of different textbook-version networks, thereby visualizing relative differences in NOS co-occurrence structures across textbook versions. These difference networks were used for descriptive comparison rather than inferential statistical testing; therefore, no statistical significance tests were conducted for the difference networks. Analysis of the difference networks (Figs 9–12) helps to summarize the commonalities and differences among the four textbooks in their representation of NOS.

As shown in Fig 9, the difference between PEP1 and PEP2 is not particularly pronounced. Although the two textbooks cover different thematic content, their overall patterns of NOS organization remain broadly comparable. PEP1, which mainly presents physical geography content, shows stronger relative connections within the cognitive-epistemic system, especially between Scientific Knowledge and Scientific Practice. This pattern is consistent with the organization of physical geography content around geographical concepts, principles, and processes, together with scientific practices such as observation, measurement, experimentation, and simulation of the Earth’s surface system.

By contrast, PEP2, which contains more human geography content, shows stronger relative connections involving social-institutional dimensions, particularly Social Values and Social Power Structures. These connections are consistent with the greater emphasis of human geography content on human-environment relationships, resource allocation, spatial governance, and the social application of geographical knowledge. Consequently, geographical knowledge in PEP2 is more often connected with social development, public decision-making, and the practical use of geographical knowledge in real-world contexts.

As shown in Fig 10, the difference between HNEP1 and HNEP2 is more pronounced than that between PEP1 and PEP2. HNEP1 is more strongly characterized by cognitive-epistemic connections, particularly those linking Scientific Knowledge, Scientific Practice, and Cognitive Aims and Values. By contrast, HNEP2 shows stronger connections involving social-institutional dimensions, especially Social Values, Social Power Structures, and Social Organization and Interaction, as well as more visible links between these dimensions and Scientific Practice. This contrast is consistent with differences in the thematic and textual organization of physical- and human-geography content within the HNEP series.

As shown in Fig 11, HNEP1 displays a more prominent “Scientific Knowledge-Scientific Practice-Cognitive Aims and Values” triangular structure than PEP1, indicating that this textbook places stronger emphasis on the internal relationships among geographical knowledge, practical activities, and disciplinary cognitive values in the organization of physical geography content. By contrast, PEP1 shows closer connections between Scientific Practice and Social values, indicating a stronger textual linkage between practical activities such as observation, experimentation, and recording and the applied value of geographical knowledge in real-world problem explanation and social life.

As shown in Fig 12, compared with HNEP2, PEP2 strengthens the connection between Scientific Practice and Methods and Methodological Rules, indicating that it places greater emphasis on presenting geographical research methods, analytical procedures, and methodological rules through practical activities in human geography content. By contrast, HNEP2 shows stronger integration among Scientific Knowledge, Scientific Norms, and Cognitive Aims and Values. This pattern indicates that HNEP2 more frequently presents geographical knowledge together with normative requirements of scientific inquiry and the cognitive value orientations underlying scientific activity.

## 6. Discussion

The Results establish both the distributional profile and the relational structure of NOS representation across the selected textbooks. The Discussion interprets these findings in relation to the three research questions by examining the distributional pattern of NOS, the organization of relationships among its dimensions, and the educational implications that can be inferred for scientific literacy and ESD-oriented geography textbook design. It then situates the Chinese textbook case within a broader international context, identifies the principal gaps in current NOS representation, and considers corresponding pathways for textbook and curriculum reform. The final section clarifies the scope and limitations of the study and outlines directions for future research.

### 6.1. Interpreting the distributional pattern of NOS representation (RQ1)

With respect to RQ1, the results reveal a clear concentration of NOS representations in the cognitive-epistemic system: 863 of the 1,196 coded NOS instances (72.2%) belong to this system, compared with 333 (27.8%) in the social-institutional system. Within this overall pattern, Scientific Knowledge and Scientific Practice are particularly prominent, although the distribution varies across the four textbooks. PEP1 and HNEP1 are organized mainly around physical-geography processes, whereas PEP2 and HNEP2 contain more human-geography content. These differences in disciplinary content provide an important context for interpreting why particular NOS dimensions receive more sustained attention than others.

Within the cognitive-epistemic system, Scientific Knowledge (C.SK) and Scientific Practice (C.SP) were the most frequently represented dimensions. Chiappetta and Fillman [23] similarly found substantial attention to scientific inquiry in U.S. high school biology textbooks. A comparable imbalance has also been reported in Chinese high school physics textbooks, where empirical NOS was particularly prominent, whereas social and sociocultural aspects received relatively limited representation [44]. Taken together, these findings suggest that uneven emphasis across NOS dimensions is not unique to geography textbooks.

The prominence of C.SK and C.SP can be interpreted in relation to the curricular and textual organization of the analyzed textbooks. In the physical geography volumes, explanations of Earth-system processes are frequently accompanied by observation, measurement, comparison, mapping, or modelling tasks. In the human geography volumes, social applications become more visible, but these applications are still commonly organized around the use of established geographical knowledge to interpret planning, development, resource, and environmental problems. Such an organization repeatedly places knowledge and practice at the center of the discourse, whereas professional communication, institutional negotiation, scientific norms, and stakeholder interaction are less often made explicit objects of exposition.

At a broader curricular level, this pattern may also be compatible with the long-standing organization of school geography around disciplinary concepts, principles, regional explanations, and observable inquiry tasks, because these forms of content can be represented explicitly in textbook exposition. Curriculum requirements and assessment practices may further reinforce attention to knowledge that can be stated, practiced, and evaluated in relatively standardized forms. However, the present study did not analyze curriculum standards or examination papers as independent datasets. These contextual factors should therefore be treated as plausible explanatory conditions rather than as causal findings of the present analysis. Future research could test them by aligning analyses of curriculum standards, textbooks, assessments, and classroom enactment.

This organization has particular significance in geography because the discipline connects natural and social scientific perspectives and frequently applies knowledge to human-environment problems [45]. Geography textbooks therefore have distinctive opportunities to connect evidence and methods with governance, policy, resource management, environmental planning, and other socially situated forms of decision-making. The relatively limited presence of Professional Activities (S.PA), Scientific Norms (S.SN), and Social Organization and Interaction (S.SO.I) indicates that these connections are only partially developed in the analyzed texts. Reinisch and Fricke [21] reported a comparable tendency in school biology textbooks, suggesting that the challenge of representing science as a socially organized enterprise extends beyond geography.

Two less visible dimensions help specify what remains implicit in the frequency profile. Social Power Structures (S.PS) vary sharply across the four textbooks, even though land use, resource distribution, environmental governance, and regional development recur as geographical concerns. Cognitive Aims and Values (C.A.V) are also less explicit than established knowledge and practical activity, despite the relevance of accuracy, skepticism, explanatory adequacy, and rational judgment to geographical inquiry. The frequency profile is therefore informative not only in terms of how much NOS content appears, but also in showing which aspects of geographical knowledge-making receive sustained emphasis in textbook discourse.

### 6.2. How textbook discourse organizes NOS relationships (RQ2)

RQ2 shifts the analysis from the amount of NOS content to its organization. The ENA topology indicates two different modes of representation: cognitive-epistemic dimensions repeatedly form a knowledge-production sequence centered on practice, epistemic aims, and established knowledge, whereas social-institutional categories enter the discourse more episodically through specific applications, organizations, or policy contexts. This distinction helps explain why frequency alone would not fully characterize the NOS profile of the textbooks.

Within the cognitive-epistemic system, Cognitive Aims and Values, Scientific Practice, and Scientific Knowledge constitute the core triadic structure in most textbooks. This configuration can be interpreted as a recurring knowledge-production script in which practical engagement is connected to established geographical knowledge and oriented by epistemic aims such as accuracy, explanation, and evidential adequacy. The representative geological-time example presented in the Results illustrates this pattern: observation and comparison of strata and fossils are linked with methodological reasoning and the construction of geological knowledge within the same local textual window. The strong C.A.V-C.SP-C.SK connections therefore reflect more than the frequent occurrence of three categories; they show that the textbooks often organize geographical inquiry around the movement from evidence-related practice toward disciplinary explanation and knowledge construction.

Methods and Methodological Rules (C.M.MR), however, remain relatively peripheral within this structure. Although methods are often embedded in activities, the textbooks less frequently make explicit why a particular method is selected, how alternative methods might produce different evidence, what assumptions constrain a method, or how methodological choices affect the strength of a geographical claim. In other words, procedural action is more visible than the methodological rationale for why a procedure is appropriate. This distinction helps explain why Scientific Practice can be strongly connected to Scientific Knowledge even when C.M.MR remains comparatively weak.

The social-institutional network is organized differently. Social values (S.SV) are often linked with Scientific Practice, especially in passages on environmental monitoring, planning, disaster response, and other applications of geographical knowledge. Connections among Professional Activities, Scientific Norms, Social Organization and Interaction, and Social Power Structures are much less frequent. Applied content is commonly presented as a sequence in which geographical knowledge or technology is introduced and then linked to practical or social value, while the institutional processes through which knowledge is negotiated, evaluated, coordinated, or contested receive less attention. The GIS-based urban-management example in the Results illustrates this pattern: geospatial practice is connected with social value and governmental application, yet interactions among agencies or stakeholders, the normative principles guiding decisions, and the weighing of competing interests and values are not represented. The relative weakness of the social-institutional system is therefore evident in its relational structure as well as in its frequency.

The difference networks reinforce this interpretation. Textbooks with stronger physical geography content, such as PEP1 and HNEP1, concentrate more strongly on connections among Scientific Knowledge, Scientific Practice, and Cognitive Aims and Values, whereas PEP2 and HNEP2 provide more opportunities for Social values and Social Power Structures through human-geography topics. However, even in the latter textbooks, social-institutional dimensions do not consistently form dense connections with one another. This suggests that simply adding policy, planning, or social-application content is insufficient. A more integrated representation would connect geographical evidence and methods with the organizations that produce or use knowledge, the norms that regulate scientific and professional practice, the interests and values involved in decisions, and the institutional structures through which action is coordinated.

### 6.3. Educational implications for scientific literacy and ESD-oriented geography textbook design (RQ3)

At the textbook level, the findings reveal an uneven pattern of representation. Geographical concepts, evidence-related practices, and epistemic aims are frequently connected in the textbook discourse, whereas their relationships with professional norms, organizational interaction, power relations, and socially negotiated applications are much less visible. Because this study focuses on textbook content rather than classroom implementation or student learning, the educational significance of this pattern is interpreted in terms of the opportunities and limitations provided by the textual representation itself, rather than as evidence of actual learning outcomes.

This pattern is relevant to scientific literacy. Kolstø [46] argues that scientific literacy for citizenship includes the resources needed to deal with the scientific dimension of controversial socio-scientific issues. Textbook design consistent with this view would make visible how geographical claims are generated and justified, together with the ways knowledge is communicated, evaluated, contested, and applied in institutional and public contexts. Weak connections between Professional Activities, Scientific Norms, Social Organization and Interaction, Social Power Structures, and the cognitive-epistemic system leave these aspects correspondingly less developed in the available textbook discourse.

This pattern has particular implications for Education for Sustainable Development. Sustainability problems combine questions of knowledge with ethical and political challenges [9]. Evidence can be uncertain, methods or models may support different interpretations, and proposed actions can distribute benefits, costs, risks, and responsibilities differently across groups and places. Textbook discourse that moves directly from geographical knowledge and scientific practice to useful applications, with little attention to institutional arrangements, stakeholder interests, social norms, competing values, and power relations, tends to present science in instrumental terms as a technical resource for an already defined problem. Less visible in such accounts are the processes through which problems are framed, acceptable responses are negotiated, affected interests are identified, and evidence is considered alongside values in public decision-making.

The same concern applies to geographical issues such as climate adaptation, water-resource allocation, land-use planning, environmental protection, disaster governance, and urban development. Geographical evidence can inform decisions in each of these areas, yet it rarely determines a single course of action. Institutional responsibilities, policy priorities, local livelihoods, environmental values, economic interests, and spatial equity can support different judgments about what should be done. Social relevance is therefore present in the textbooks, as the comparatively visible Social values dimension indicates, but the processes through which socially relevant geographical knowledge is debated, coordinated, and translated into collective action receive more limited treatment.

For ESD-oriented textbook design, increasing the number of social-institutional references would address only part of the problem. Their connections with cognitive-epistemic dimensions also need to be strengthened. A textbook task might ask what evidence supports a geographical claim, how that evidence was produced, which methodological assumptions or uncertainties remain, which organizations and stakeholders are involved, what norms or responsibilities apply, and how alternative values or interests shape possible decisions. Such tasks would keep the analysis grounded in disciplinary geography while making links among evidence, methods, values, institutions, and action more explicit. The relevant textbook-level goal is a wider and more connected representation of NOS, without assuming that this representation produces a direct or automatic effect on learner outcomes.

The design issue is therefore one of connection. Isolated references to institutions, ethics, policy, or social value leave the overall organization of NOS largely unchanged. Greater change would come from linking these elements directly with the evidence, methods, practices, and knowledge claims already prominent in geography content. Textbook discourse could then show more clearly how geographical knowledge moves from inquiry and explanation into professional, institutional, and public settings. This would broaden the textual resources available for NOS and ESD-oriented reasoning while remaining within the limits of what textbook analysis can establish about student learning.

### 6.4. Chinese textbook case and broader international implications

The present analysis concerns selected Chinese senior secondary compulsory geography textbooks. These materials also offer a case through which broader questions about geography education can be considered. Developed under national curriculum standards, they show one way in which scientific-literacy and sustainability-oriented aims are translated into teachable content. The results draw attention to the structural relations among geographical knowledge, inquiry practices, social values, and institutional contexts, in addition to the selection of textbook content itself.

In an international context, the Chinese case highlights a recurring design problem in geography education: curriculum materials need to retain the discipline’s strong treatment of knowledge and inquiry while making the social organization and public use of that knowledge more explicit. This concern is especially relevant to geography because the discipline connects natural and social sciences and deals with complex human-environment problems. An ESD-oriented representation would therefore attend to how geographical claims are generated, validated, communicated, contested, and applied when institutions, stakeholders, and competing values enter real-world decision-making.

The study contributes to international discussion in several connected ways. Extending FRA-based NOS analysis to geography brings a discipline with strong interdisciplinary and sustainability-oriented characteristics into a literature dominated by conventional science subjects. Combining FRA with ENA also makes it possible to examine the relational structure of NOS dimensions in curriculum materials alongside their frequency. For textbook development, teacher education, and ESD-oriented curriculum reform, the findings point to the value of connecting cognitive-epistemic and social-institutional dimensions so that relations among evidence, methods, values, institutions, and action are more explicit. These connections are relevant to public reasoning around science-related issues [46] and to ESD approaches that emphasize reflection and engagement with sustainability challenges [47].

The analytical approach may also be useful for textbook studies in other national contexts. Future work could compare how geography curricula organize NOS and sustainability-related content across countries and examine how particular textbook representations are enacted in classrooms and interpreted by learners. Evidence of this kind is needed before textbook-level opportunities can be linked empirically with outcomes in scientific literacy or sustainability-oriented reasoning.

### 6.5. Addressing the gaps: pathways for reform

The findings suggest that textbook and curriculum reform should give greater attention to how NOS dimensions are made explicit and connected. Numerical balance across FRA categories is less informative than the extent to which the epistemic core of geographical content is linked with the professional, normative, organizational, and institutional conditions under which geographical knowledge is produced and used.

Curriculum guidance could state NOS learning goals more clearly across the dimensions of FRA. Professional Activities, Scientific Norms, Social Organization and Interaction, and Social Power Structures are relatively weak in the analyzed textbooks. Guidance at the curriculum level could therefore give more explicit attention to the social organization of scientific work, ethical norms, institutional contexts, power relations, and the role of scientific knowledge in sustainability-related decision-making, alongside geographical knowledge and scientific practice.

Textbook development could also integrate NOS dimensions more systematically across physical and human geography topics. Short prompts, inquiry tasks, or reflective questions embedded in disciplinary content can show how geographical knowledge is generated, what evidence and methods support it, how uncertainty is handled, how claims are debated and applied, and how social groups, institutions, values, and interests enter decision-making. This form of integration would present geography more explicitly as a scientific enterprise situated in social contexts without necessarily requiring substantial increases in textbook length.

Teacher professional development is another relevant part of this process. Because social-institutional dimensions are less visible in the analyzed textbooks, professional learning can help teachers identify places where institutional contexts, ethical norms, social values, or sustainability-related decision processes remain implicit. Geography teacher education and in-service training could include NOS-focused modules based on frameworks such as FRA, enabling teachers to extend textbook materials through questions, discussion, and reflection while distinguishing clearly between content provided by the textbook and content introduced through instruction.

Assessment alignment deserves attention at the wider curriculum-system level. An assessment system centered almost exclusively on factual recall may leave less curricular space for the epistemic and social dimensions of geographical knowledge. Future assessment tasks could ask learners to evaluate evidence, compare methodological approaches, identify uncertainty, examine the social use of geographical knowledge, and reason about sustainability-related problems. The present study did not analyze examinations or student performance, so this point is offered as a direction for curriculum development and future research rather than as an empirical result of the current analysis.

### 6.6. Limitations and future research directions

Several limitations define the scope of the present findings. The analysis is confined to textbook content and does not address classroom implementation. Future research could examine how teachers use these materials, which NOS elements they make more explicit or leave unaddressed, and how students interpret the resulting instruction. Classroom observation and student interviews would provide evidence on these processes.

The sample consists of four mainstream Chinese senior secondary compulsory geography textbooks. These books are appropriate for examining NOS representation at the compulsory senior secondary level, but the findings cannot be generalized to all geography textbooks used in China. Additional textbook versions, elective modules, and international samples would allow the scope and generalizability of the findings to be examined more fully.

The analysis also relies on a nine-dimensional FRA-based framework and ENA. This combination permits examination of both the distribution and relational structure of NOS dimensions, but other coding frameworks may reveal different aspects of textbook representation. Longitudinal work could examine changes in NOS representation across curriculum reforms, while design-based studies could develop and test supplementary materials or instructional strategies aimed at strengthening social-institutional dimensions in geography education.

## 7. Conclusion

Using the Family Resemblance Approach and Epistemic Network Analysis, this study examined NOS representation in selected Chinese senior secondary geography textbooks in the context of sustainable development. Across 1,196 coded instances, Scientific Knowledge and Scientific Practice account for much of the frequency profile. ENA adds a relational view: Cognitive Aims and Values, Scientific Practice, and Scientific Knowledge form a recurrent core; Methods and Methodological Rules occupy a more peripheral position; and Social values most often enter the networks through the practical application of geographical knowledge. Professional Activities, Scientific Norms, Social Organization and Interaction, and Social Power Structures are less consistently integrated into these knowledge-practice relations.

In the context of sustainable development, the findings are significant for the relationships that textbook discourse makes visible among geographical knowledge, evidence, practices, values, and institutional contexts. The analyzed materials provide substantial textual opportunities for engaging with the cognitive-epistemic dimensions of geographical knowledge, while the professional organization, normative regulation, social negotiation, and institutional application of such knowledge receive more limited treatment. This distinction matters for sustainability-related topics because evidence and methods can inform decisions without determining how competing values, interests, responsibilities, and policy options are ultimately reconciled.

The study extends FRA-based textbook analysis to geography and shows how ENA can be used to examine the relational structure of NOS representations alongside their frequency. For ESD-oriented geography textbook design, the findings point to the need for stronger connections between social-institutional elements and geographical knowledge, evidence, methods, practices, and real-world decision contexts. Such integration would broaden the textual opportunities for representing geography as an epistemic practice situated within social and institutional settings.

These conclusions apply to the selected textbook corpus. Classroom implementation, teacher mediation, assessment effects, and students’ actual NOS understanding were outside the scope of the analysis, so no direct claims are made about learning outcomes. Future research can connect textbook analysis with classroom observation, teacher practice, student data, additional textbook versions, and international comparison to examine how different forms of NOS representation are enacted and interpreted in educational settings.

## Supporting information

S1 File Original coded dataset. The complete coded dataset used for the quantitative analyses and figure generation.
(XLSX)

S2 File Sampled dual-coder reliability dataset. The sampled dual-coder reliability dataset used to assess inter-coder reliability.
(XLSX)
